# A UK Healthcare Professional Perspective on the Implementation of Monitoring in Children and Young People With Early‐Stage Type 1 Diabetes: A Qualitative Study

**DOI:** 10.1111/dom.71141

**Published:** 2026-07-26

**Authors:** Rabbi Swaby, Julia Townson, Rachel E. J. Besser

**Affiliations:** ^1^ Diabetes and Inflammation Laboratory, Centre for Human Genetics, Nuffield Department of Medicine, Bukhman Centre for Research Excellence in Type 1 Diabetes, NIHR Oxford Biomedical Research Centre University of Oxford Oxford UK; ^2^ Centre for Trials Research Cardiff University Cardiff UK; ^3^ Department of Paediatrics University of Oxford Oxford UK

**Keywords:** early‐stage, management, monitoring, staging, type 1 diabetes

## Abstract

**Aims:**

Research studies that screen and monitor children and young people (CYP) with early‐stage type 1 diabetes (T1D) are gaining momentum, but the views of healthcare professionals (HCP) on the ability to implement monitoring strategies in clinical practice remain unclear. This study aims to explore the perspectives of HCPs in the United Kingdom on monitoring CYP with early‐stage T1D in clinical care.

**Materials and Methods:**

Fifteen HCPs across the United Kingdom took part in a semi‐structured interview, with representation from across the paediatric diabetes multidisciplinary team. The perspectives of HCPs on monitoring CYP with early‐stage T1D for progression to clinical disease were explored. Data analysis was conducted using a thematic analysis approach.

**Results:**

Three themes were generated: (1) Uncertainty managing the early stages of T1D; (2) The need for guidance, training and consistent messaging; (3) Integrating monitoring into clinical care. Participants highlighted that knowledge gaps created uncertainty in their approach to monitoring. HCPs sought guidance and clear pathways to support their decision‐making on understanding the risk of progression associated with early‐stage T1D and which test to use when monitoring and communicating risk to families. HCPs were supportive of monitoring within clinical care but emphasised the need for expansion of clinical services to meet increased caseloads and upskilling the HCP workforce.

**Conclusions:**

HCPs would support the monitoring of CYP within a clinical care setting but highlight important barriers to implementation that need addressing before national rollout. This includes HCP education, clear pathways to ensure national guidance and standardisation of clinical practice, and expansion of clinical services.

## Twitter/X Summary

UK healthcare professionals support monitoring children and young people with early‐stage type 1 diabetes, highlighting knowledge gaps and the need for clear guidance and expanded services before national rollout. #T1D #DiabetesResearch #Paediatrics.

## 1 Introduction

Screening for islet autoantibodies to detect the early stages of type 1 diabetes (T1D) is gaining international momentum, with a growing number of research programmes being developed worldwide [1, 2]. Early‐stage T1D is categorised into glycaemic stages, with stage 1 and 2 defined by the development of two or more islet autoantibodies, with accompanying normoglycaemia (stage 1) or dysglycaemia (stage 2), and stage 3 by persistent islet auto‐immunity and hyperglycaemia, with or without symptoms [3, 4]. Following the identification and confirmation of multiple islet autoantibodies, individuals require metabolic staging and monitoring. This monitoring period, which can last months or years, has been shown to be an essential component of screening programmes, in order to see the clinical benefits associated with screening [5], namely, prompt recognition of evolving hyperglycaemia, early intervention with insulin therapy to prevent diabetic ketoacidosis [6, 7, 8] and the ability to offer disease‐modifying therapies to delay clinical onset.

As the identification of children and young people (CYP) with early‐stage T1D increases, there is growing recognition that the psychosocial and practical implications of monitoring require further exploration [9]. To date, most research has focused on screening, reporting that healthcare professionals (HCPs) generally support screening, but both HCPs and parents have concerns about causing psychological harm, including increased health anxiety among families [10, 11, 12, 13]. Studies have also highlighted uncertainty among HCPs when managing early‐stage T1D and the practical implementation of screening within clinical care [10, 11].

In contrast, relatively little is known about the delivery of monitoring, including initiation of insulin, after the identification of early‐stage T1D [9, 14]. One study conducted in Australia used semi‐structured interviews to explore HCP perspectives on the implementation of a monitoring programme and reported a need for additional education and training to support clinical delivery [15]. Recently, a national survey of UK HCPs identified substantial variation in monitoring practices, suggesting a need for clearer guidance and clinical pathways [16]. As monitoring becomes increasingly integrated into clinical care, understanding how it can be delivered effectively and acceptably is essential. Therefore, this study explored the perspectives of paediatric diabetes HCPs on the acceptability of a national T1D monitoring programme in the UK, and potential barriers to its implementation.

## 2 Materials and Methods

### 2.1 Study Design

This qualitative study used semi‐structured interviews to explore UK HCP perspectives on monitoring CYP with early‐stage T1D. We adopted this approach because it provided flexibility for the researcher to probe and explore with the participant, opinions and perspectives that were outside of the interview schedule.

### 2.2 Participants and Recruitment

Between March and August 2025, we interviewed 15 HCPs, recruited via an advert distributed across professional clinical networks around the UK (England and Wales), including the British Society for Paediatric Endocrinology and Diabetes and the National Paediatric Diabetes Network. HCPs were included if they had been involved in the delivery of monitoring for at least one CYP with early‐stage T1D. We developed a purposive sampling framework to ensure representation from at least one doctor (consultant), one nurse specialist, one dietician and one psychologist from the paediatric diabetes multidisciplinary team (MDT) [17].

HCPs provided informed consent (completed remotely via Microsoft Teams) after reviewing the study information provided, prior to their interview. This study was granted ethical approval from the South Birmingham Research Ethics Committee (reference: 23/WM/0184, 15 July 2024) and was registered with the UK Health Regulation Authority.

### 2.3 Data Collection

We developed a semi‐structured interview schedule with clinicians and methodologists (Data S1). This semi‐structured approach ensured that the discussion remained relevant to the study aims, while allowing the interviewer to probe and explore specific perspectives raised by the participant. The topics explored included: participants' background, experience of delivering monitoring and integration of monitoring into clinical care (Table 1, Data S1).

**TABLE 1:** Outline of key topic areas explored during interviews.

Background
Participants' role, years of experience and clinical background Experience of managing clinical T1D, early‐stage T1D and personal experience of T1D
Experience of delivering monitoring
Participants' views on who should deliver monitoring, including the setting Views on the test choice and frequency of testing Perspectives on what support would be needed for HCPs to deliver monitoring
Integration of monitoring into clinical care
Perspectives on the integration of monitoring in current clinical services Anticipated challenges and consideration for how current HCPs would implement monitoring Exploration of what expertise, information or training would be needed to prepare HCPs

Abbreviations: HCP, healthcare professional; T1D, type 1 diabetes.

R.S. conducted the interviews using Microsoft Teams, which were audio recorded and transcribed using the built‐in transcription function [18]. R.S., an experienced paediatric doctor, undertook qualitative training from an experienced qualitative researcher (J.T.) before conducting the interviews as part of their PhD qualification. R.E.J.B. is a consultant paediatric endocrinologist with expertise in diabetes and has led the delivery of an early‐stage T1D clinic, supporting clinicians across the UK to implement monitoring. In contrast, J.T. is a researcher with an interest in the early diagnosis of T1D but has no clinical experience of T1D monitoring. Although a formal reflexivity framework was not used, the differing clinical and research backgrounds within the team facilitated discussion and consideration of alternative interpretations during analysis. Three participants were known professionally to R.S.

Interviews lasted on average 44 min, range (30–66 min). RS reviewed, corrected and anonymised all transcripts, which were then uploaded, managed and coded using NVivo15 software [19]. Data collection and analysis were conducted concurrently, to allow emerging findings to be explored in subsequent interviews, through revision of the interview schedule (Data S1).

### 2.4 Data Analysis

We used an inductive thematic analysis approach to promote the exploration of participants' experiences and opinions, while minimising any potential bias, by reflecting and balancing our interpretation of the data [20]. This process included iterative stages: (1) familiarisation; (2) coding; (3) initial theme generation; (4) review and development of themes; (5) refinement of theme names and definitions.

R.S. and J.T. independently coded interview data for the first three interviews (20% of transcripts), to compare matched codes, resolve differences and to consider whether the interview schedule needed to be adjusted. No further interviews were conducted until consensus was reached, and R.S., J.T. and R.E.J.B. had agreed on a coding framework that captured key features of the data relevant to the research question. R.S. used this framework to guide subsequent coding of the remaining interviews. R.S. then generated initial themes supported by key quotations, which were then further refined through discussion with R.S., J.T. and R.E.J.B., to develop the finalised themes and subthemes [21].

## 3 Results

The study sample included 15 paediatric HCPs (six consultant paediatricians, five nurses, two dieticians and two psychologists) from 13 different paediatric diabetes units (six tertiary centres, seven district general hospitals). Additional demographics are outlined in Table 2. Here, we report the perspectives of HCPs involved in the monitoring of CYP with early‐stage T1D, relating to their experiences on current clinical monitoring within the UK National Health Service, their views on the support HCPs need to deliver monitoring, the potential for a future national monitoring programme within clinical care, and barriers to implementation.

**TABLE 2:** Summary of participant characteristics.

Characteristics	*n*
Total number of interviewees	15
Gender	
Male	3
Female	12
Ethnicity	
White	12
Black	1
Asian	2
Mixed	0
Other	0
Multidisciplinary team role	
Total number of interviewees	15
Paediatric diabetes consultant	6
Paediatric diabetes nurse specialist	5
Paediatric diabetes dietitian	2
Paediatric psychologist	2
Diabetes experience, years	
< 5 years	6
5–10 years	3
> 10 years	6
Type of healthcare setting	
Tertiary hospital	6
District general hospital	7

### 3.1 Thematic Analysis: Healthcare Professionals' Perspectives on the Implementation of Monitoring Children and Young People With Early‐Stage T1D

**FIGURE 1:**
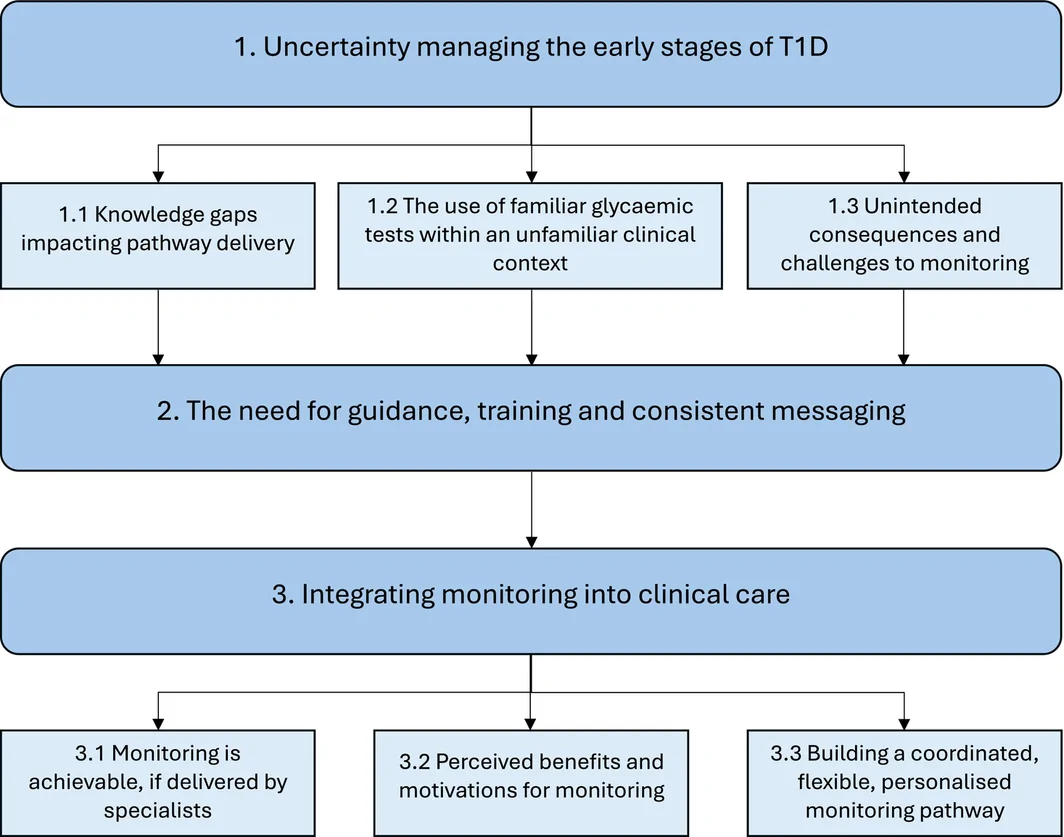
A top‐down flow diagram outlining the key topics identified.

Three themes were identified: (1) Uncertainty managing the early stages of T1D; (2) The need for guidance, training and consistent messaging; (3) Integrating monitoring into clinical care (see Figure 1). The themes were organised into two overarching concepts, perceived barriers and challenges to implementation, which included theme 1, and motivations and enablers of monitoring, which included themes 2 and 3. Supportive quotes that illustrate our findings have been collated into their respective themes (Table 3).

**TABLE 3:** Quotes to support each outlined theme.

1. Uncertainty managing the early stages of T1D
1.1 Knowledge gaps impacting pathway delivery
‘I don't know if how high the antibodies are, makes any difference’ (P1, Doctor)
‘In this situation, it's the uncertainty in contrast to all the other kids with type 1 about what, what changes in glucose and HbA1c are significant enough to say this now needs intervention with insulin’ (P6, Doctor)
‘So it's been seven months and it's been a very different kind of way of management and we've not, have we done it very well? I'm not sure. Because I found there's been a lot, because we've been not, we've not been quite sure how things are going to go. We've not been quite sure how to manage things’ (P12, Nurse)
‘It's really hard because you want to get a balance of not freaking people out by making them more really, really anxious and also not wanting to, you know, create lots of behavioural changes’ (P2, Psychologist)
‘So regularly above 7 in the morning, we'd start Tresiba, regularly above 10, 10 and above post meals. We'd start NovoRapid’ (P13, Doctor)
‘He didn't start on any basal insulin and has been like that for two years. We've had other young people who've started on basal, but no NovoRapid, so it seems a bit as if we don't really know quite what the right thing to do is’ (P9, Nurse)
‘I had prescribed everything that they potentially would need at home just because again, we weren't sure of how quick things could develop’ (P5, Nurse)
‘… they're probably safer at home just starting on tiny doses with the nurses, liaising with me … about doses’ (P13, Doctor)
1.2 The use of familiar glycaemic tests within an unfamiliar clinical context
‘I suppose, the HbA1c from a diagnostic point of view is helpful’ (P4, Dietician)
‘The point of care testing is done by our nurses. We don't have to approve it with anyone else’ (P1, Doctor)
‘Yeah, I think it depends on how fast they're progressing, doesn't it? For the ones that are progressing really quickly, it probably lags a little bit behind.’ (P6, Doctor)
‘I think something that you know, has robust meanings to the answers it gives you, to the results, so like an OGTT’ (P6, Doctor)
‘Does it make any difference at all if OGTT is high? If the blood sugars are not high persistently on CGM or fingerpricks?’ (P7, Doctor)
‘[CGM] gives you a better, more information and it saves the children having to prick their fingers that often, and we're quite comfortable looking at CGM numbers’ (P10, Doctor)
‘CGM is useful and helpful and paints a picture. But if you gave 10 different people a CGM at the moment you'd get 10 different answers about what it means’ (P6, Doctor)
‘Yeah. So they will get, we give them all blood glucose and ketone testing to keep them safe’ (P13, Doctor)
1.3 Unintended consequences and challenges of monitoring
‘But it was really hard because they were low carbing her…’ (P6, Doctor)
‘He wasn't going to school as much as there was worries within school, because his [CGM] alarm was going off in school’ (P12, Nurse)
‘everything's been heightened up to this point, but because we don't have a particular pathway to follow, we don't have. It's uncharted territory for us, really’ (P12, Nurse)
‘I've done, there's been a lot, a lot of support. So then we're thinking, […] was it even worth it?’ (P12, Nurse)
2. The need for guidance, training and consistent messaging
‘Yeah, I think kind of some good guidance and policies around when to introduce insulin, what insulin to introduce, whether it's just you know, at what point do you just introduce basal?’ (P3, Nurse)
‘What I would want to ensure is that every consultant within the team we had a consensus as to what we would offer’ (P1, Doctor)
3. Integrating monitoring into clinical practice
3.1 Monitoring is achievable, if delivered by specialists
‘Yes. I think it can. I think it might need a bit of an overhaul. Of how it happens. If, like you say, the numbers zoom up, I think right now I feel it's manageable and it's fine. It depends what happens going forwards in terms of how many people’ (P3, Nurse)
‘I think we've no choice but to do that. And then as numbers might rise, will rise, then yeah, we just have to go through the process of what happens when anything like that happens’ (P6, Doctor)
‘I think they [diabetes team] are the best people to do it. However, I don't think we've necessarily got the capacity to do it’ (P8, Dietician)
‘In an ideal world, it would be the diabetes team. The diabetes team would be able to answer questions about what's to come’ (P1, Doctor)
‘GPs see kids with diabetes so infrequently. We often have kids who've been referred, who've come in, having seen their GP a couple of times already with properly developing type 1, and GPs haven't picked up on it’ (P9, Nurse)
‘If they had, you know, sort of guidelines to follow and things and how often I feel like, yeah, that would be less likely for it, for it to get missed’ (P4, Dietician)
3.2 Perceived benefits and motivations for monitoring
‘Not wanting them to drop out, but wanting to prevent a kind of DKA and more acute kind of diagnosis’ (P2, Psychologist)
3.3 Building a coordinated, flexible, personalised monitoring pathway
‘Yeah. Yeah, that we don't want to kind of over medicalise it or create too much burden or stress for them or any more than is needed’ (P2, Psychologist)
‘Once they were in like stage 3, then it might be helpful to see them maybe face to face and meet the team that will event will go on to be caring for them as a way of kind of easing them into that transition to a full diagnosis’ (P2, Psychologist)
‘…if they had three antibodies and they were under five, I think I'll be safe, I'll feel it's better to be looked after by the hospital’ (P10, Doctor)
‘We don't really do virtual appointments. I think because you need an HbA1c even for the pre‐type ones because most of them are having three monthly HbA1c’ (P13, Doctor)

Abbreviations: CGM, continuous glucose monitoring; GP, general practitioners; HbA1c, glycated haemoglobin; OGTT, oral glucose tolerance test.

### 3.2 Perceived Barriers and Challenges to Implementation

#### 3.2.1 Uncertainty Managing the Early Stages of T1D

This theme represented the experiences of HCPs as they attempted to navigate the management of CYP with early‐stage T1D. HCPs described their current understanding of early‐stage T1D, the nuances of decision making when considering how to approach monitoring, and their concerns about the challenges of monitoring and causing unintended harm to their patients. It included three subthemes: (1.1) Knowledge gaps impacting pathway delivery; (1.2) The use of familiar glycaemic tests within an unfamiliar clinical context; (1.3) Unintended consequences and challenges of monitoring.

##### 3.2.1.1 Knowledge Gaps Impacting Pathway Delivery

This subtheme reflected the current understanding of early‐stage T1D among HCPs, and the challenges they experienced in navigating the monitoring process and communicating risk with their patients. HCPs reported a lack of understanding of the definition of early‐stage T1D, and the risk factors associated with progression to clinical disease. This lack of knowledge was reflected in their uncertainty on how to assess for disease progression. As a result, HCPs lacked clarity on the overall approach to monitoring, including choice and frequency of testing. Clinicians described their difficulty in not knowing what would happen next, hampering their ability to plan a monitoring strategy and communicate the risk associated with early‐stage T1D. Many HCPs expressed doubt on how best to ensure families were appropriately safety‐netted without causing additional anxiety and harm.

There were differing levels of confidence reported by HCPs in relation to the introduction of insulin therapy. Some HCPs reported a clear plan with defined glucose thresholds that would warrant intervention, while others reported that there was a lack of rationale to support team decision making on when to intervene and what type of insulin to use. In some instances, not only did HCPs demonstrate a clear plan for when insulin therapy would be started, but they also initiated treatment at home, opting not to admit the CYP to hospital. Instead, HCPs provided outpatient education and training to prepare the family over time.

##### 3.2.1.2 The Use of Familiar Glycaemic Tests Within an Unfamiliar Clinical Context

HCPs described the nuanced decision‐making process behind glycaemic testing outside of their clinical experience of managing stage 3 T1D. There was variation in the tests used to monitor CYP with early‐stage T1D among participants. There was also uncertainty around how the use of these tests differs when interpreting them outside the context of clinical T1D. Tests used included the HbA1c, continuous glucose monitoring (CGM), the oral glucose tolerance test (OGTT) and at‐home glucose and ketone monitoring.

Participants described the HbA1c as useful when it can support a clinical diagnosis and preferred to use the test in line with their previous experience. Participants also described the HbA1c as easier to perform, with fewer logistical barriers compared to other tests, as it can be performed at the point of care. However, there were some participants who demonstrated knowledge of the use of the HbA1c in the context of early‐stage T1D monitoring. These participants expressed concern when using the HbA1c in those who could progress to stage 3 quickly, suggesting that it could miss these individuals.

The OGTT was also described as a helpful test because it supported clinical decision making and gave clear results on a patient's glycaemic status. However, participants questioned whether results from the OGTT should supersede those from tests that give information over time, such as self‐monitored glucose testing or CGM. Specifically, participants highlighted the benefits of CGM, given it can provide large amounts of real‐time glucose data. HCPs were also familiar with using CGM as part of their day‐to‐day practice in managing CYP with clinical T1D. Despite this preference and familiarity, there was also uncertainty when using CGM for CYP with early‐stage T1D, who were not on insulin therapy. This resulted in an inconsistency in how the test was used, such as different thresholds to determine abnormality and consequently, interpretation of CGM results.

Despite the different approaches to test use in the monitoring of early‐stage T1D, there was some recognition of the need for safety netting, anticipating that some individuals could progress between monitoring visits. Monitored individuals were often offered home blood glucose and ketone monitors and given instructions on when to test and contact their local diabetes team.

##### 3.2.1.3 Unintended Consequences and Challenges of Monitoring

This subtheme encompassed the challenges and trade‐offs of monitoring that gave HCPs cause for concern. In principle, participants supported monitoring. However, they also recognised that it could inadvertently cause health anxiety and create uncertainty for HCPs tasked with delivering monitoring in the absence of clear guidance. Some participants described unforeseen effects of monitoring that gave them cause for concern. These included parents offering less carbohydrates to their children. Participants also reported that some CYP experienced disruption to their education, as CGM alarms were causing anxiety within the school environment.

In addition to the concerns described about the impact on the CYP and their families, the process of monitoring also presented challenges for the HCPs involved. The apparent lack of guidance left participants unclear if they were providing good quality care. This lack of clarity, coupled with the increased workload, appeared to be a demotivating factor for some individuals who questioned whether they were doing the right thing to monitor the CYP.

### 3.3 Motivations and Enablers of Monitoring

#### 3.3.1 The Need for Guidance, Training and Consistent Messaging

In addition to reporting the current challenges faced when delivering monitoring, in this theme, HCPs also outlined the factors that could be addressed to alleviate some of these uncertainties and enable them to deliver monitoring. Most HCPs reported that good quality guidance would be an important factor in supporting them to deliver monitoring and reassure clinicians that there was a benefit to monitoring CYP in the presymptomatic stage of disease, rather than at the onset of clinical symptoms. Further, HCPs felt additional education would provide an improved basic understanding of early‐stage T1D and help HCPs gain insights into what is expected of them. HCPs outlined several ways in which additional education could be made accessible, including teaching at regional meetings and webinars. Some HCPs felt a consensus was important to ensure consistent messaging with patients. Finally, HCPs reported that it would be important for clinicians to see good quality evidence for monitoring to support their clinical practice.

#### 3.3.2 Integrating Monitoring Into Clinical Practice

This theme encompassed participants' perspectives on the acceptability of monitoring for early‐stage T1D, who should deliver this service, the perceived benefits and motivations for monitoring and how this could be implemented to develop robust, patient‐centred clinical pathways. This comprised three subthemes: (3.1) Monitoring is achievable, if delivered by specialists; (3.2) Perceived benefits and motivations for monitoring; (3.3) Building a coordinated, flexible, personalised monitoring pathway (Figure 1).

##### 3.3.2.1 Monitoring Is Achievable, if Delivered by Specialists

This subtheme described the views of HCPs on whether monitoring could be achieved in UK clinical care, and the need to protect individuals at risk of progression to clinical disease. Nearly all HCPs reported that in their professional view, at current levels, monitoring for early‐stage T1D within the UK healthcare setting was possible. However, they reported that an expansion of current services would be needed, especially if the number of individuals being monitored were to significantly increase. Some HCPs felt a duty to provide monitoring, describing it as a moral obligation given the knowledge that CYP are being identified without symptoms.

In addition to the overall perspective that monitoring was possible, all HCPs reported that CYP with early‐stage T1D should be monitored by a diabetes service within the hospital setting. HCPs described the rationale for monitoring to be led by paediatric diabetes specialist teams. Despite concerns around workload and resource provision, HCPs felt the diabetes MDT had the necessary experience, expertise and access to diabetes technology to provide this service. Most HCPs reported concern when asked about alternative settings as potential monitoring locations, such as primary care. HCPs felt the professionals within this setting did not have the necessary experience to safely and efficiently provide monitoring in this context, and specific pathways would be needed to make this option feasible. Ultimately, HCPs felt that any queries would eventually come to their team, meaning the use of alternative pathways would not necessarily result in reduced workload for the local diabetes team. Finally, HCPs felt that given the long‐term nature of T1D, it would be important to give patients a positive start and engage with the diabetes team from the outset of their clinical journey.

##### 3.3.2.2 Perceived Benefits and Motivations for Monitoring

Participants gave their views on why monitoring should be offered to CYP with early‐stage T1D. Participants viewed monitoring as a preventative intervention, not because it altered disease progression, but because it reduced the risks associated with delayed diagnosis and disengagement from clinical care. This included the prevention of diabetic ketoacidosis, which was described as a key reason to monitor, or to prevent an acute presentation. Some participants demonstrated an understanding of current issues within early‐stage T1D monitoring, including high attrition rates, which they cited as a good reason to monitor to keep their patients engaged.

##### 3.3.2.3 Building a Coordinated, Flexible, Personalised Monitoring Pathway

To deliver monitoring, HCPs described the need for system‐level infrastructure to support monitoring, rather than relying on individual efforts. This included the creation of robust clinical pathways to ensure clear lines of communication and levels of responsibility, especially when both a DGH and tertiary centre are involved in a CYP's care.

Minimising invasiveness and disruption for those monitored was seen as an important factor in reducing burden on the families. HCPs suggested methods to optimise monitoring, offering strategies that would support ongoing engagement. HCPs felt that offering flexible monitoring options, with combinations of face‐to‐face and virtual clinics, would be a useful way to support families. In this context, HCPs suggested that formalising monitoring as patients approached stage 3 T1D was a gentler way to transition into clinical T1D care. This would also help to familiarise the families with the clinical setting and the diabetes team, which could help them feel more prepared. In addition, this would allow them to provide additional support outside of glycaemic testing, such as advice on healthy eating. HCPs reported that frequent monitoring may not be necessary, and instead, a risk‐stratified approach should be used to determine the frequency of monitoring. Finally, HCPs emphasised the need for treatments that can be offered during the early stages of T1D to support the justification for early identification and monitoring.

## 4 Discussion

Our study is the first to explore the views and experiences of paediatric diabetes HCPs involved in the monitoring of CYP with early‐stage T1D in the UK and it is one of only two in‐depth interview studies on this topic globally [15]. HCPs viewed monitoring within the UK healthcare system as acceptable while outlining challenges that would need to be addressed before the UK is ready for the implementation of a widespread screening and monitoring programme. HCPs highlighted the existing high workload experienced by paediatric MDTs, acknowledged that fundamental knowledge gaps created uncertainty in managing CYP with early‐stage T1D, and stressed the importance of education to improve the experience of HCPs practising within a field that is developing rapidly.

### 4.1 Monitoring Is Possible With Careful Planning

Our finding that HCPs were supportive of a potential future monitoring programme is similar to a recent survey of family paediatricians in Italy by Mari et al., who reported that 84% expressed a willingness to participate in a T1D screening programme [11]. In contrast, a recent study in the UK reported that professional stakeholders were in support of research into screening but expressed caution regarding general population screening, given a lack of evidence on public acceptability [10]. In our study, HCPs felt that hospital‐based diabetes teams should lead on the delivery of future monitoring, given the current limited role of primary care doctors in T1D management for CYP in the UK. This is different to the healthcare models used in other countries, where family paediatricians work within primary care, playing an important role in German and Italian screening and monitoring [22, 23]. The high workload and constrained resources are significant barriers to the implementation of monitoring within the UK healthcare setting, similar to findings identified by Ospelt et al. [12]. The need for a treatment to justify screening and monitoring identified in our study is also supported by Mari et al., who described concerns at the lack of definitive treatment options, and Quinn et al., who described research into general population screening as a priority, should an immunoprevention agent be licensed in the UK, which has since occurred [10, 13]. HCPs across these studies report similar views that careful planning would be required to implement a national programme [11, 12].

HCPs in our study expressed uncertainty in the understanding of early‐stage T1D, which tests would be useful when monitoring, and how to initiate insulin therapy. This is consistent with the findings of a recent UK survey that found a lack of consistency in the approach to monitoring by clinical teams [16]. Mari et al. also reported that HCPs lacked knowledge of early‐stage T1D [11], and Majstorovic et al. described HCP uncertainty when initiating insulin therapy [15]. These findings may represent a general uncertainty within the current literature, given there is no consensus on the approach to insulin initiation, and the optimal choice of glycaemic test during monitoring remains under debate [1, 14, 24, 25]. The ambiguity described in these studies may stem from an unawareness of available guidance and processes to support HCPs less involved with early‐stage T1D. For example, Mari et al. found that only 24% of respondents felt sufficiently informed of the national screening programme in Italy, and 73% did not feel adequately prepared to fulfil their role in its delivery [11]. Our findings indicated that most HCPs were unaware of the available guidance on this topic, with a few participants demonstrating knowledge of the ISPAD guidelines [26], but none referencing the Breakthrough Type 1 international consensus [25]. The few participants that understood early‐stage T1D and the related guidance were predominantly doctors.

HCPs in this study expressed concern about causing unintended harm when discussing the risks associated with early‐stage T1D, describing a tension between adequate safety netting and inducing unwanted behaviour change in their patients. Similar findings were reported by Majstorovic et al., stating that HCPs were concerned with how best to communicate with families, and Mari et al. also reported concerns about behaviour change in families, which has been demonstrated in the minority of screened CYP [11, 15, 27]. Similarly, Quinn et al. reported parental concerns about inducing health anxiety among CYP and their families [10, 13]. In contrast, Ospelt et al. reported that the majority (96%) of HCPs felt comfortable discussing T1D risk and islet autoantibody results with patients [12]. It might be that these HCPs had more awareness of early‐stage T1D, with several stating their involvement with the TrialNet study [28].

Our study offers unique insight to the perspectives of HCPs on the monitoring of CYP with early‐stage T1D within the UK healthcare system. The only prior qualitative interview study with HCPs focussed on their educational needs in delivering monitoring, highlighting important knowledge gaps that would need to be addressed [15]. Our study expanded on this, highlighting a lack of clinical experience among HCPs in monitoring early‐stage T1D. This included uncertainty in monitoring strategies, navigating discussions on the implications of an early‐stage diagnosis and the initiation of insulin therapy. Despite these challenges, HCPs were in support of monitoring and provided suggestions on how barriers to implementation could be overcome. These included HCP training and support of clinical services.

### 4.2 Strengths and Limitations

This study provides valuable insights into the acceptability of a future monitoring programme, from the perspective of paediatric diabetes MDT members within the UK healthcare setting. However, our sample did not include primary care practitioners (also known as General Practitioners) working in the community setting. Primary care practitioners are a main access point to healthcare in the UK, but current practice limits their role in paediatric T1D [29]. Our study included 15 HCPs, and the findings should not be generalised to all HCPs across the UK. Further, there is the potential for self‐selection bias within our sample, as those with a vested interest in early‐stage T1D monitoring may be more inclined to take part in studies seeking their views. Our sample included a spread of different paediatric MDT members, with equal representation from tertiary and district general hospital settings to mitigate this.

### 4.3 Implications

Our findings indicate a need for accessible, actionable guidance to allow HCPs to deliver monitoring for CYP. The process of upskilling the current workforce will be an important step to ensure successful implementation. Recently, the British Society for Paediatric Endocrinology and Diabetes published best practice guidance on the screening and monitoring of CYP for early‐stage T1D in the UK [30]. This provides the foundation for the standardisation of practice, development of structured education, and creation of local guidance. Further, it is likely that services will need to be adapted to support the increased workload as monitoring increases. In some cases, this might lead to expansion of diabetes services or separate pathways streamlined for early‐stage T1D care. Table 4 provides a roadmap to the implementation of a monitoring programme within clinical care, with areas identified by HCPs that require further consideration.

**TABLE 4:** Summary of recommendations to support the implementation of clinical monitoring.

Training, education and support for HCPs
Development of accessible information and training packages to support continued professional development. Dissemination of guidance to support management strategies, and assessment of impact, to inform future iterations of best practice recommendations. Coordination of training and guidance through national networks and professional bodies.
Supporting the implementation of monitoring
Creation of pathways that minimise patient and HCP burden while maximising utility. Learning from current practice, collaboration with current ‘Early‐T1D’ clinics with regional centres to optimise monitoring approaches. Ongoing clinical data collection to allow impact analysis of screening and monitoring on clinical outcomes at the population level.
Cost implications associated with monitoring
Cost‐effectiveness analysis to include healthcare professional workload and monitoring pathway.

Abbreviations: HCP, healthcare professional; T1D, type 1 diabetes.

### 4.4 Future Work

Further research is needed to better understand the practical and cost implications of implementing screening and monitoring at a national level. Given the inconsistent awareness of pre‐existing monitoring guidelines [25, 30], an assessment of the impact of the latest UK guidance (released after data collection for this study) on clinical practice is also needed.

Future work should also focus on improving our understanding of the perspectives of CYP and their parents/families, and their experience of testing. Given that monitoring can extend over several years, this will form an important part of any attempts to coordinate widespread screening and monitoring.

The implementation of monitoring within the UK healthcare setting is acceptable to clinicians and will require HCP training and support, alongside an expansion of current services. We highlight the need for robust evidence to support monitoring, clear pathways to standardise practice and structured education to support HCPs.

## Funding

This work was supported by the Novo Nordisk UK Research Foundation (Novo Nordisk UK Research Fellowship 2022).

## Conflicts of Interest

R.S. has received speaker honoraria from Sanofi. R.E.J.B. reports receiving speaker honoraria from Eli Lilly and Springer Healthcare, reports sitting on the Novo Nordisk UK Foundation Research Selection Committee on a voluntary basis, acting as an independent advisor for Provent Bio, and received speaking honoraria from Sanofi and Medscape, which were donated to an education research fund. The other author declares no conflicts of interest.

## Supporting information


**Data S1:** The topic guide used to explore healthcare professionals' perspectives on monitoring during semi‐structured interviews.

## Data Availability

The data that support the findings of this study are available from the corresponding author upon reasonable request.
