# Supplementary material for: A UK Healthcare Professional Perspective on the Implementation of Monitoring in Children and Young People With Early‐Stage Type 1 Diabetes: A Qualitative Study
Source: Diabetes Obes Metab. 2026 Jul 26;28(10):9326–35. doi: 10.1111/dom.71141 (PMC13538742; doi:10.1111/dom.71141)
Supplement: Supplementary file 1 [file DOM-28-9326-s001.docx]

**SUPPLEMENTARY MATERIAL**

**Interview schedule for healthcare professionals**

The questions in this interview schedule (topic guide) are intended to be a guide and not definitive. The themes that we wish to explore will be highlighted in **BOLD** for clarity.

This interview schedule is semi-structured to allow the interviewer to explore other topics that may arise during the interview. This process is intended to be iterative, so future interviews may be adapted based on information from earlier interviews.

**INTRODUCTION:** (to be read verbatim to the participant)

Thank you for agreeing to speak with me today. My name is XXX and I am a researcher at the XXXXX. The research that I do involves learning more about children and young people who have diabetes antibodies, who may need insulin in the future. I am interested in what happens after they have been identified with these antibodies, and how the process of being followed up affects them and their families. I am keen to hear your experiences, views and opinions of children and young people being monitored for the development of type 1 diabetes, and how this may or may not be implemented into NHS clinical care in the future.

I would like to hear about your experiences, views and opinions, in your own words. There are no right or wrong answers, as anything you tell me will be useful. Even if there is something you don’t agree with, that would be really helpful, to allow us to plan for the future. Any information you provide will be anonymous so you can be completely honest.

If you need to stop the interview at any time, that is absolutely fine. Can I check that you have read the Participant Information Sheet? Do you have any questions before we start?

I am now going to read out the statements on the consent form, please answer yes or no after each statement to confirm if you agree.

***Read out consent form.***

**INTRODUCTORY/PREAMBLE QUESTIONS**

1) Could you tell me about your role as a healthcare professional?

***Prompts: how long they have worked in that role, where they have worked, have they had other roles in the past that are related.***

2) Could you tell me about any personal experience you have of type 1 diabetes, if any, relating to you or a family member?

***Prompts: Do they or does anyone else in their family have diabetes, if so what type, does that influence how they view diabetes.***

3) Could you tell me about your experience of managing type 1 diabetes, including before insulin has been started?

***Prompts: How many patients have they seen with pre-T1D, what help did they need if any, who did they seek advice from, how did they decide when to start insulin, did they involve their diabetes MDT.***

**VIEWS ON MONITORING QUESTIONS**

1) Could you tell me who you think should be responsible for monitoring these individuals?

***Prompts: Should it be primary care, or diabetes professionals, or someone else. Probe – why they chose that option.***

2) What impact do you think it would have if the individual were monitored in primary care, or in school?

***Prompts: What role would secondary care play, what support might be needed.***

3) How often do you feel these individuals should be seen?

***Prompts: Should it be as regular as diabetes patients, more or less. Probe – why they gave that answer.***

4) What support might be needed for healthcare professionals seeing children and young people in the early stages of type 1 diabetes?

***Prompts: What type of support would be helpful, guidelines, seminars, CPD sessions, joint care with a tertiary or quaternary service.***

5) What tests do you feel would be appropriate when monitoring a child or young person for T1D?

***Prompts: What tests have they used in the past, what tests do they feel most comfortable interpreting. Probe – are there any other reasons for the options(s) chosen.***

6) What other support do you feel they would need, and do you feel the diabetes MDT are able to give that support?

***Prompts: What support do they think these individuals would need, would that be different to those with T1D, could their MDT support those needs.***

**INTEGRATION INTO CLINICAL CARE QUESTIONS**

1) Can monitoring children for type 1 diabetes be integrated into clinical care?

***Prompts: Their general feelings for the future, are these views impacted by possibility of type 1 diabetes affecting their child.***

2) Could this be incorporated into your current services?

***Prompts: Probe – why they feel that way, what has influenced that feeling.***

3) Do you anticipate any staffing changes would be needed to support this service?

***Prompts: Could the current staff manage, would there need to be more of a particular member of the MDT.***

4) What expertise, information or training would be needed to help staff prepare for this service?

***Prompts: Do staff already have some experience of these cases, if no experience what forum would they usually look to for information, support or training.***
